# Beyond the Scent: New Evidence about Micromorphological, Phytochemical and Biological Features of *Plumeria rubra* ‘Tonda Palermitana’ (Apocynaceae)

**DOI:** 10.3390/plants13172479

**Published:** 2024-09-04

**Authors:** Paola Malaspina, Mariarosaria Ingegneri, Federica Betuzzi, Emilio Di Gristina, Laura Cornara, Domenico Trombetta, Antonella Smeriglio

**Affiliations:** 1 Department of Earth, Environment and Life Sciences (DISTAV), University of Genova, Corso Europa 26, 16132 Genova, Italy; paola.malaspina@unige.it (P.M.); federica.betuzzi@edu.unige.it (F.B.); laura.cornara@unige.it (L.C.); 2 Department of Chemical, Biological, Pharmaceutical and Environmental Sciences, University of Messina, Viale Ferdinando Stagno d’Alcontres 31, 98166 Messina, Italy; mariarosaria.ingegneri@unime.it (M.I.); domenico.trombetta@unime.it (D.T.); 3 Department of Agricultural, Food and Forest Sciences (SAAF), University of Palermo, 90128 Palermo, Italy

**Keywords:** *Plumeria rubra*, Pomelia, *P. rubra* flowers, *P. rubra* leaves, micromorphology, polyphenols, iridoids, terpenoids, antioxidant properties, anti-inflammatory properties

## Abstract

*Plumeria rubra* L. is an ornamental Caribbean plant widely known for its ethnobotanical uses and pharmacological activities. The ‘Tonda Palermitana’ cultivar, on which no data are to date available, is commonly cultivated in Sicily. The aim of our study was to characterize the micro-morphological features of leaves and flowers of this cultivar by light and Scanning Electron Microscopy and to investigate the phytochemical profile and the biological properties of their food-grade extracts (LE and FE, respectively) by LC-DAD-ESI-MS analysis and different in vitro assays. Numerous branched laticifers were observed, and their secretion contained alkaloids and lipophilic compounds as confirmed by histological analyses. Phytochemical analyses showed the presence of alkaloids (9%), terpenoids (13%) and fatty acids (6%), together with a very abundant presence of iridoids (28%) and polyphenols (39%). The most notable biological activity of both extracts appears to be the antioxidant one, showing half-inhibitory concentrations (IC_50_) about 5 times lower than those detected in anti-inflammatory assays (383.74 ± 5.65 and 232.05 ± 2.87 vs. 1981.23 ± 12.82 and 1215.13 ± 10.15, for FE and LE, respectively), with LE showing the best, and statistically significant (*p* < 0.001), biological activity. These results allow us to speculate promising nutraceutical and cosmeceutical applications for this old Sicilian cultivar.

## 1. Introduction

The genus *Plumeria* L. (Apocynaceae family) includes laticiferous shrubs and small trees widely cultivated as ornamental plants [1]. In this genus, hundreds of scientific names used for species, varieties and cultivars are now regarded as synonyms. According to The Plant List [2], the accepted species within the *Plumeria* genus are 11, whereas according to Plants of the World [3], there are 18. Among these, the most cultivated species are *P. obtusa* L., *P. pudica* Jacq., and *P. rubra* L. [4]. *P. rubra*, also known as ‘Frangipani’, is certainly the most used due to its beautiful and fragrant flowers of different colours and forms [1]. The first description of this plant can be found in the Badianus Manuscripts of 1522, a book written by the Spanish priest Francisco de Mendoza on the medicinal uses of local plants by the Aztec population. However, the genus name *Plumeria* was given in honour of the seventeenth-century French botanist Charles Plumier [5,6,7].

*P. rubra* is native to Central America but it has been spread across tropical and subtropical areas of the world [1]. In Hawaii, its flowers are traditionally used for the famous flower necklaces (Lei), which have become a symbol of brotherhood and hospitality [4].

In India, the plant is a symbol of immortality, and in the past, it was commonly planted near temples and graveyards [8]. The species is also cultivated in Italy, along the coastal areas of Sicily, especially in Palermo, where it has been renamed ‘Pomelia’ [4]. It is also called ‘Frangipani’, from the French word ‘frangipanier’, coagulated milk, in relation to its latex (or sap). It is not known exactly when the first plant of *P. rubra* arrived in Palermo. The first notices date from the second decade of the nineteenth century and concern the Boccadifalco Botanic Garden, which, together with the one in Palermo, seems to have constituted the nucleus of the propagation of this species in the city [4]. Since its arrival in Palermo, the plant was immediately appreciated for the beauty and intense scent of its flowers, so much so that it quickly spread in private and public gardens or was grown in containers on balconies and terraces, becoming one of the symbols of the city’s floral displays. However, in Palermo, *P. rubra* has not only ornamental use but also a profound cultural significance. It is an auspice of fertility formulated by analogy with the remarkable example of the vegetative force of the plant. Therefore, its white flowers are often used for the bouquets of Palermo brides and traditionally the bride who goes to live in a new house receives a plant from her mother as a wish for family continuity [4].

*P. rubra* is a deciduous tree of about 7–8 m, with a thin and grey-green bark and swollen branches which are leafy at the tips [1]. All the portions of the plant are characterized by the presence of a white milky latex, which can be irritating to the skin [9]. Leaves are lanceolate to oblong, having an acute base and acute to acuminate apex, while the large fragrant flowers can range from pink-red to white-yellow in colour [7].

The typical form of the plant cultivated in Palermo is characterized by a white corolla, yellow in the centre, intensely scented, with reflected round petals; it is generically named ‘Tonda Palermitana’, ‘Classica Palermitana’ or ‘Acutifolia’.

According to the literature, about 110 chemical compounds have been found in *P. rubra*. These include iridoids, terpenoids, flavonoids, alkaloids, coumarins, etc., whose presence and quantity can vary in different portions of the plants as well as in different types of extracts. Therefore, several pharmacological activities of *P. rubra* have been reported, such as antibacterial and anti-inflammatory, anxiolytic, antidiabetic, hypolipidemic and many other properties. Moreover, no mortality or signs of toxicity have been recorded for methanolic or ethanolic extracts of the leaves, pods and flowers in in vivo tests for the doses used, suggesting a high margin of safety [7].

Leaves are used in folk medicine to treat inflammations, ulcers, rheumatism, bronchitis, cold and other diseases [10]. Leaf infusions or extracts have been reported for use against asthma [11], while their decoction is employed in South America as purgative/anthelmintic or for the treatment of various skin problems [12]. Flower decoction is instead used in Mexico for the treatment of diabetes mellitus [13,14]. Used in particular doses, flowers can be useful for birth control [15]. Flowers eaten with betel leaves (*Piper betle* L.) are used as a febrifuge [16] while, together with other herbs, are part of the recipe of the ‘Five Flower Tea’, a popular cooling beverage considered as a remedy for ‘Hot Qi’ [17]. Latex is used as a good rubefacient in rheumatism [18] or topically applied on blisters and sores [19]. In addition, the petals of many *Plumeria* species and varieties are used as edible flowers in different dishes, as well as for cosmetic purposes [20].

However, no studies on the biological properties of extracts from the old Sicilian cultivar of *P. rubra* are to date available. Therefore, the aim of our study was to investigate, by a multidisciplinary approach, the macro- and micro-morphology of both the leaves and flowers of *P. rubra* ‘Tonda Palermitana’ and to evaluate the phytochemical features and biological properties of their hydroalcoholic extracts.

## 2. Results

### 2.1. Macro-, Micro-Morphological and Anatomical Studies

**Figure 1:**
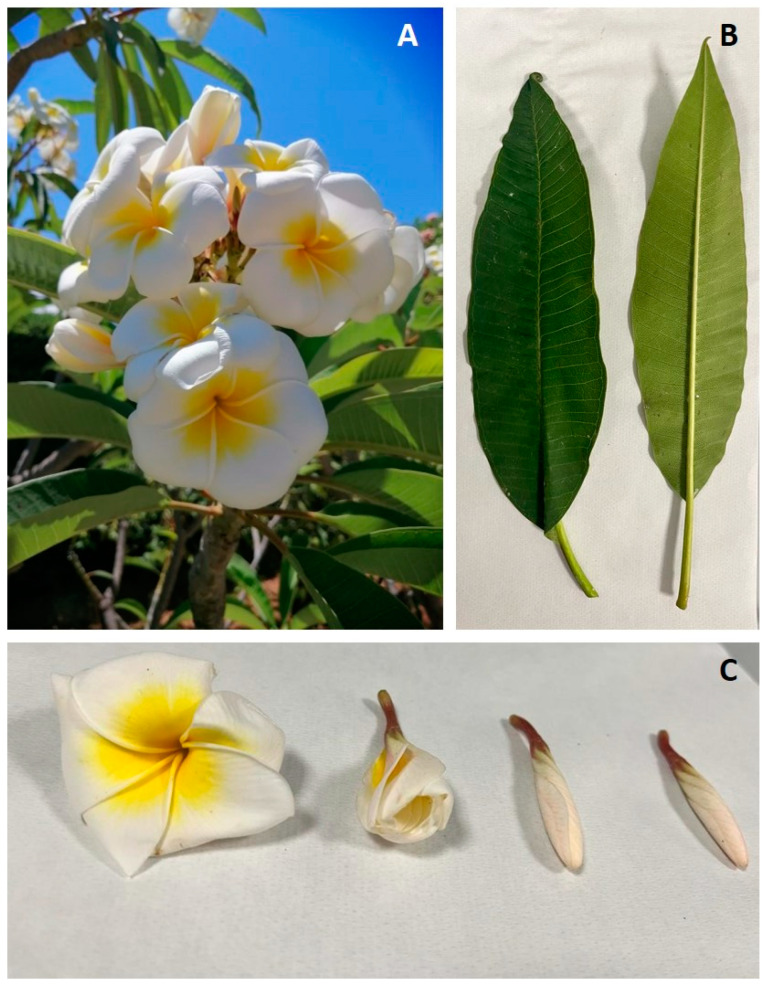
Flowers and leaves of *P. rubra* ‘Tonda Palermitana’: (**A**) plant growing in Mondello (Palermo, Italy) (photo by E. Di Gristina); (**B**) adaxial and abaxial surfaces of the leaves (photo by P. Malaspina); (**C**) flowering stages (photo by A. Smeriglio).

The main characteristic features of the plant in bloom are shown in Figure 1A–C. The leaves, up to 30 cm in length, are elliptic with an entire margin and a pointed tip. The leaf blade is bright green and leathery, with parallel secondary veins running from the midrib to the margin (Figure 1B). *P. rubra* flowers are arranged in terminal or lateral cymes. Each inflorescence consists of many white flowers with a yellow throat. The fragrant flowers show five petals fused at the base in a short funnel-shaped tube (Figure 1C).

**Figure 2:**
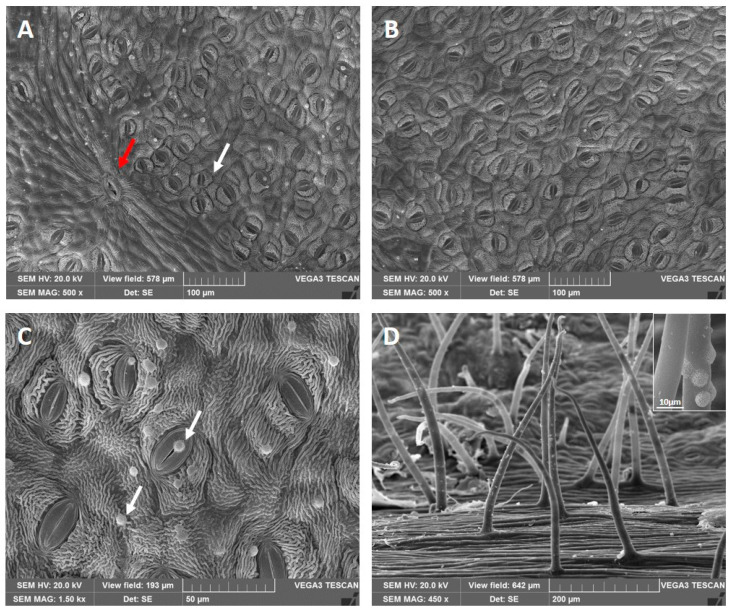
Leaf anatomy under Scanning Electron Microscopy: (**A**) adaxial surface, evidencing the paracytic stomata (white arrow) and a solitary stomata on the rib (red arrow); (**B**) stomata on the abaxial surface; (**C**) magnification of the adaxial surface showing striated cuticle and waxes forming small spheres (white arrows) scattered on both the epidermal cells and stomata; (**D**) NGTs on the ribs with a detailed view of the waxes present on their surfaces (in the right frame).

The leaves are amphistomatic and show sunken paracytic (or rubiaceous) stomata (Figure 2A white arrow and Figure 2B,C) and prominent solitary stomata slightly longer than the paracytic ones, present only on the ribs (Figure 2A, red arrow). On both leaf surfaces, the cuticle is densely striated, and waxes are arranged in small spheres scattered over the epidermis, stomata and trichomes (Figure 2C, white arrows). The leaves are glabrous except for the ribs on the lower surfaces, where uniseriate, pluricellular non-glandular trichomes (NGTs) with an acute apex are located (Figure 2D). The right frame of Figure 2D shows a magnified view of the NGTs where spherical waxes are randomly distributed.

**Figure 3:**
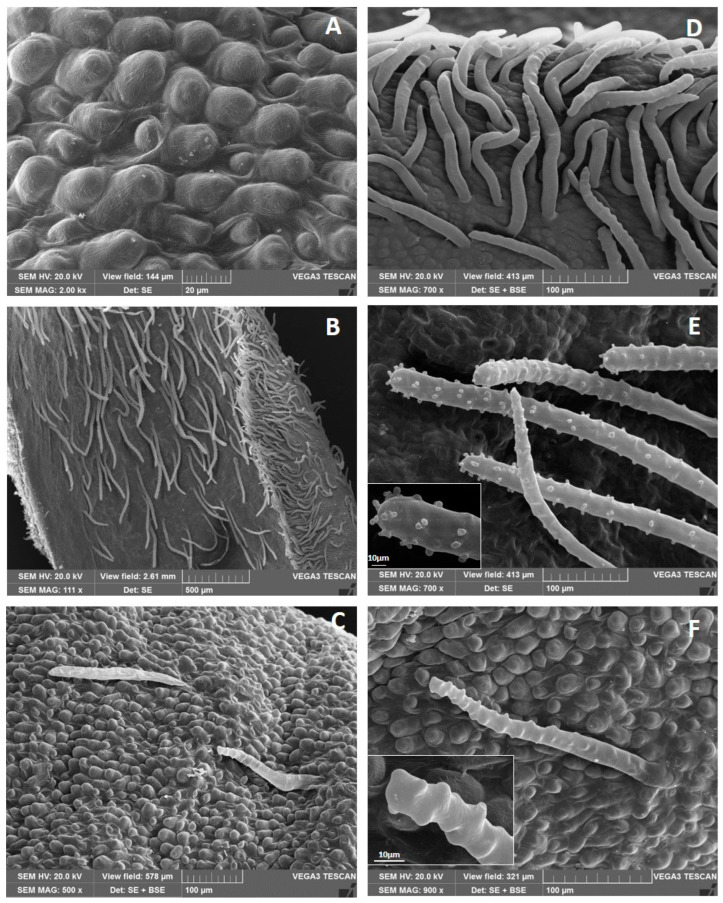
Petal anatomy under Scanning Electron Microscopy: (**A**) conical papillate cells on the outer epidermis; (**B**) basal portion of the floral funnel-shaped tube with numerous NGTs on both surfaces; (**C**) apical portion of the petal showing few NGTs on its inner surface; (**D**) NGTs on the outer surface with a smooth or slightly wavy surface and a pointed or rounded apex; (**E**) NGTs with small protuberances on their surface; (**F**) NGTs with protruding knobs. (**E**,**F**): in the left frames are visible details of the two NGTs surface.

The whole outer petal epidermis presents prominent and conical papillae (Figure 3A). The floral funnel-shaped tube is densely pubescent, showing numerous NGTs on both the inner and outer side of its basal zone (Figure 3B). However, the abundance of these trichomes decreases towards the apex of the petal (Figure 3C). Different types of unicellular NGTs are observed: (1) long, with a smooth or slightly wavy surface and a pointed or rounded apex (Figure 3D); (2) long, with an irregular surface due to the presence of small protuberances (Figure 3E) or of protruding knobs (Figure 3F). The protuberances and the knobs are clearly visible in the frame of the respective figures (Figure 3E,F).

**Figure 4:**
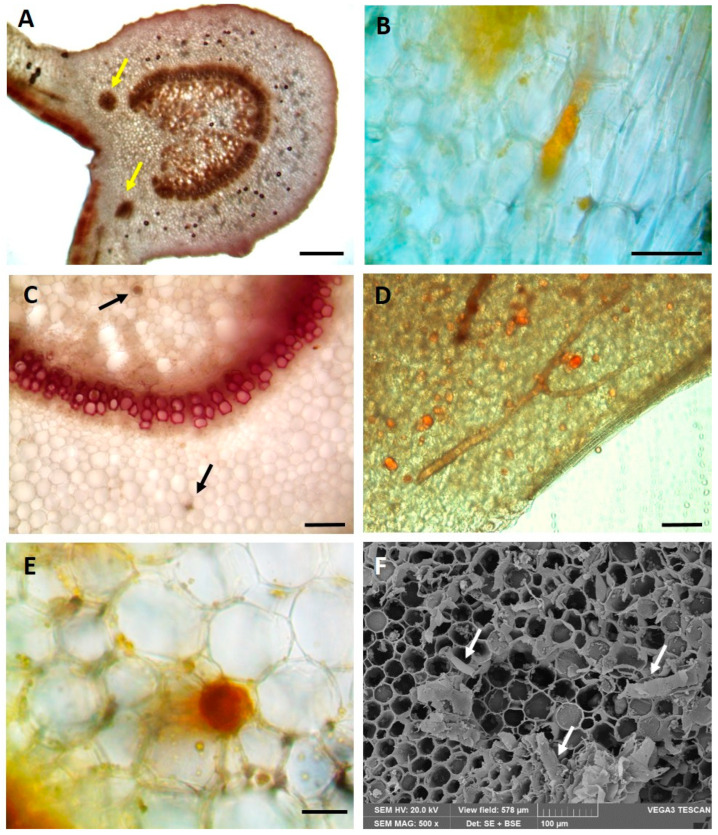
Light (**A**–**E**) and Scanning Electron Microscopy (**F**) micrographs: (**A**) leaf transversal section evidencing the midrib structure, yellow arrows indicate the two accessory bundles; (**B**) orange coloured laticifer in leaf transversal section stained with Dragendorff reagent; (**C**) leaf transversal section stained with Phloroglucinol-HCl showing the bi-collateral bundle and two laticifers (black arrows); (**D**) laticifers in petal epidermal peel reacting positively with Sudan III; (**E**) a laticifer positive to Dragendorff reagent and surrounded by radially arranged parenchymatic cells; (**F**) petiole transversal section showing small portions of laticiferous tubes (white arrows) scattered in the parenchyma. Bars: (**A**) = 200 μm, (**B**) = 50 μm, (**C**) = 100 μm, (**D**) = 100 μm, (**E**) = 50 μm.

In the transversal section, the leaves show a dorso-ventral mesophyll (Figure 4A). A bi-collateral, arc-shaped vascular bundle accompanied by two smaller accessory vascular bundles at each side is visible in the midrib (Figure 4A, yellow arrows). Additionally, in all examined organs, numerous branched laticifers are present. They are observed in the leaves (Figure 4B,C, black arrows) and petals (Figure 4D) and in the transversal section of the petioles, where they are detected by both light microscopy (Figure 4E) and Scanning Electron Microscopy (Figure 4F, white arrows). In this latter case, they appear as small portions of laticiferous tubes. Both in the leaf and in the petiole, the laticifers are irregularly dispersed in the parenchyma, occurring close to the phloem on either side of the vascular cylinder (Figure 4C, black arrows). In the transversal section, they appear polygonal or circular (Figure 4C, black arrows) and enclosed in a rosette of parenchymatic cells (Figure 4E). The latex reacted positively with the Dragendorff reagent (Figure 4B,E), indicating the presence of alkaloids, and with Sudan III staining (Figure 4D), showing the presence of lipophilic compounds.

### 2.2. Phytochemical Analyses on Food-Grade Extracts

After a careful macro and micro-morphological analysis, food grade extracts (FE and LE, respectively) were prepared using an ethanol/water mixture (80:20, *v*/*v*) obtaining a good and reproducible extraction yield (7.59% ± 0.27 and 8.69% ± 0.43 for FE and LE, respectively).

The phytochemical profile was first investigated by several colorimetric assays aimed at quantifying the main classes of secondary metabolites such as total phenols, total flavonoids, flavan-3-ols, as well as other flavonoids that have a free meta-oriented hydroxy group on the B ring (vanillin index), and proanthocyanidins. This also allowed us to establish the polymerization degree (vanillin index/proanthocyanidins), which if it is higher than 1 shows a greater abundance of monomer molecules. These results are shown in Table 1.

LE was the richest extract not only in terms of total phenols but also in terms of flavonoids, with statistically significant differences (*p* < 0.05) with respect to FE. On the contrary, no statistically significant difference was detected in terms of proanthocyanidins content. Both extracts show a predominance of monomeric molecules according to the high polymerization index detected, with LE showing the best value according to the vanillin index results (Table 1).

This preliminary screening was followed by an in-depth phytochemical characterization by LC-DAD-ESI-MS analysis (Table 2). Compounds were detected and tentatively identified by comparison of mass and UV–Vis spectra with literature data and online free consulting spectra databases, as well as with commercially available reference standards (see Table 2 and relative footnote for details).

A total of 54 compounds belonging to the classes of iridoids (31%), flavonoids (19%), phenolic acids (17%), terpenoids (13%), alkaloids (9%), fatty acids (6%) sterols (4%) and coumarins (2%) were identified, with 35 in FE and 34 in LE (Table 2).

These results confirm what was previously detected by Dragendorff reagent and Sudan III staining in micro-morphological analyses, which is the presence of alkaloids and lipophilic compounds (see Section 2.1) such as terpenoids, fatty acids and sterols (Table 2). This is also consistent with the results of the preliminary phytochemical screening, which also highlighted the abundant presence of flavonoids and phenolic acids (Table 1).

**Figure 5:**
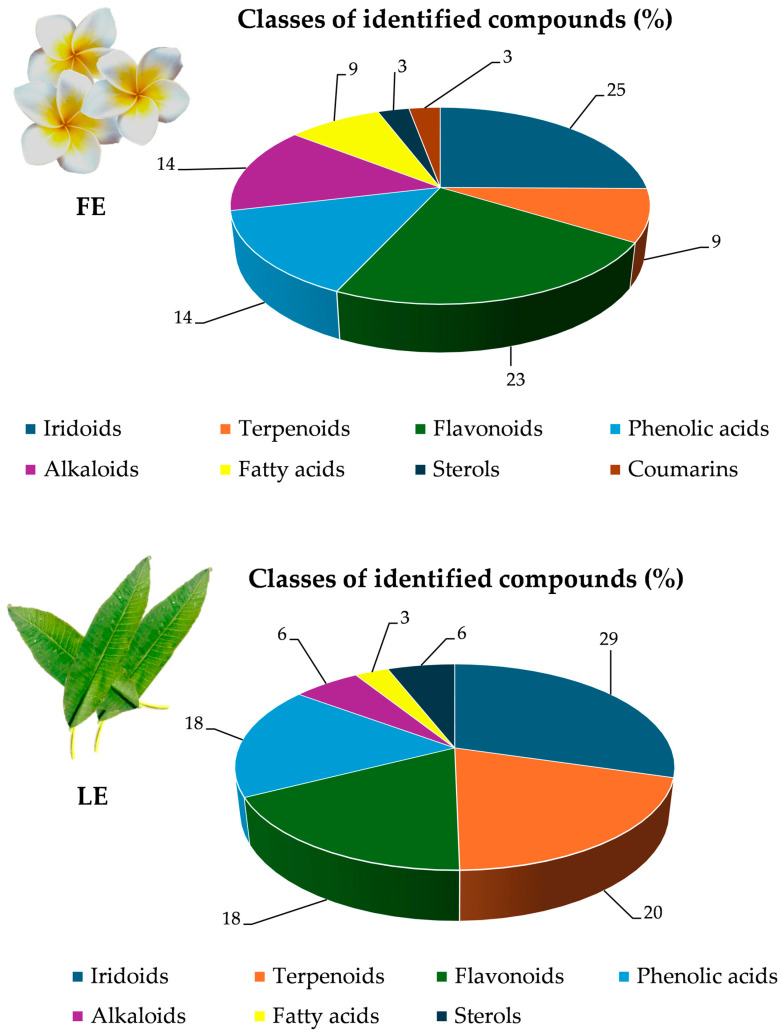
Distribution percentage of phytochemical classes identified in *P. rubra* ‘Tonda Palermitana’ flower and leaf extracts (FE and LE, respectively).

Expressing the phytochemical data of FE and LE in terms of percentage distribution of the various classes of detected compounds, it is immediately evident that the two extracts show substantial differences. Indeed, as depicted in Figure 5, apart from the iridoid class, which remains almost unchanged, in percentage terms, in the two extracts under examination (25% and 29% in FE and LE, respectively), FE showed the highest content of flavonoids (23% vs. 18% in LE), alkaloids (14% vs. 6% in LE) and fatty acids (9% vs. 3% in LE). Furthermore, FE contains the coumarin scopoletin, which it did not find in LE.

On the contrary, LE showed the highest content of terpenoids (20% vs. 9% in FE), phenolic acids (18% vs. 14% in FE) and sterols (6% vs. 3% in FE).

**Figure 6:**
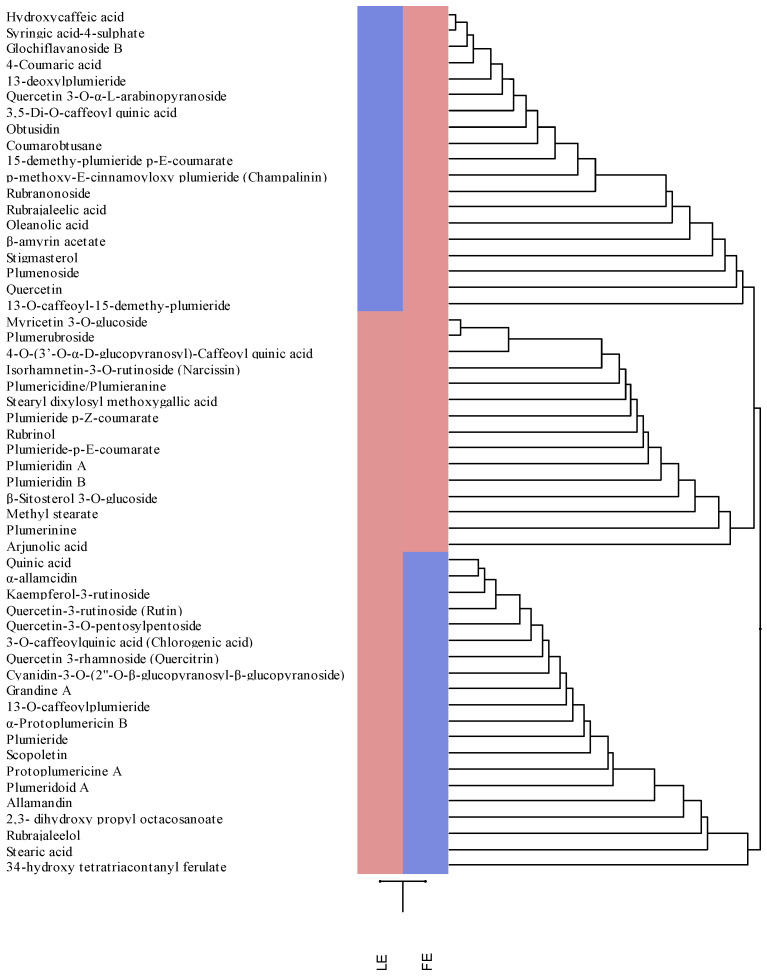
Agglomerative hierarchical clustering analysis of the phytochemical data of *P. rubra* ‘Tonda Palermitana’ flower and leaf extracts (FE and LE, respectively) obtained by LC-DAD-ESI-MS analysis. The heatmap shows the expression pattern of the identified metabolites, indicating in red and blue the most and the least expressed metabolites, respectively. Colour density indicates the fold change between the investigated extracts.

The agglomerative hierarchical clustering analysis allowed us to delve deeper into the phytochemical differences found between the two extracts under examination (Figure 6). Since a qualitative analysis was carried out in the present study, a 0 score was assigned for the not found metabolites and a 1 score for those found in the extracts under examination. The analysis returns a heatmap that identifies the presence of the metabolite with the red colour and its absence with the blue colour. This allows for a quick and easy comparison of the LE and FE phytochemical profiles.

As shown in Figure 6, three clusters were found, of which two (the first and the last one from the top to the bottom) identify the secondary metabolites present in LE and absent in FE, and the third (in the middle) identifies the 15 metabolites common to LE and FE. Specifically, these are the iridoids plumieride *p*-Z-coumarate, plumieride-*p*-E-coumarate, plumieridin A and plumieridin B; the flavonoids myricetin 3-*O*-glucoside, isorhamnetin-3-*O*-rutinoside (narcissin) and plumerubroside; the phenolic acids 4-*O*-(3′-*O*-α-D-glucopyranosyl)-caffeoyl quinic acid and stearyl dixylosyl methoxygallic acid; the terpenoids rubrinol and arjunolic acid; the alkaloids plumericidine/plumieranine and plumerinine; the fatty acid methyl stearate and the sterol β-Sitosterol 3-*O*-glucoside.

### 2.3. Biological Properties

The iron-chelating activity (ICA), the anti-peroxidase properties by measuring the β-carotene bleaching (BCB) inhibition, and the free-radical scavenging properties of FE and LE against several charged radicals, such as 2,4,6-tris(2-pyridyl)-s-triazine (tptz) for ferric reducing antioxidant power (FRAP), 2,2-diphenyl-1-picrylhydrazyl (DPPH), 2,2′-azino-bis(3-ethylbenzothiazoline-6-sulfonic acid) (ABTS) for trolox equivalent antioxidant capacity (TEAC) and 2,2′-azobis(2-methylpropionamidine) dihydrochloride (AAPH) for oxygen radical absorbance capacity (ORAC), were investigated by several in vitro spectrophotometric and spectrofluorimetric assays based on different mechanisms and reaction environments. Furthermore, the ability of FE and LE to inhibit the heat-induced bovine serum albumin denaturation (ADA) and the protease activity (PIA) was investigated to evaluate the anti-inflammatory properties of FE and LE.

The results, which were expressed as half-inhibitory concentration (IC_50_) with the respective confidence limits at 95%, are shown in Table 3.

According to the quantitative phytochemical results (Table 1), LE showed the lowest half-inhibitory concentration (IC_50_) values and therefore the strongest antioxidant and anti-inflammatory activity in all the assays carried out, although statistically significant results (*p* < 0.05 vs. FE) were observed only in the DPPH, ICA and ADA tests (Table 3).

Calculating the average of the IC_50_ values, the most notable biological property of both extracts appears to be the antioxidant one, showing IC_50_ values about 5 times lower than those detected in anti-inflammatory assays (383.74 ± 5.65 and 232.05 ± 2.87 vs. 1981.23 ± 12.82 and 1215.13 ± 10.15, for FE and LE, respectively), with LE showing the best, and statistically significant (*p* < 0.001), biological activity.

**Figure 7:**
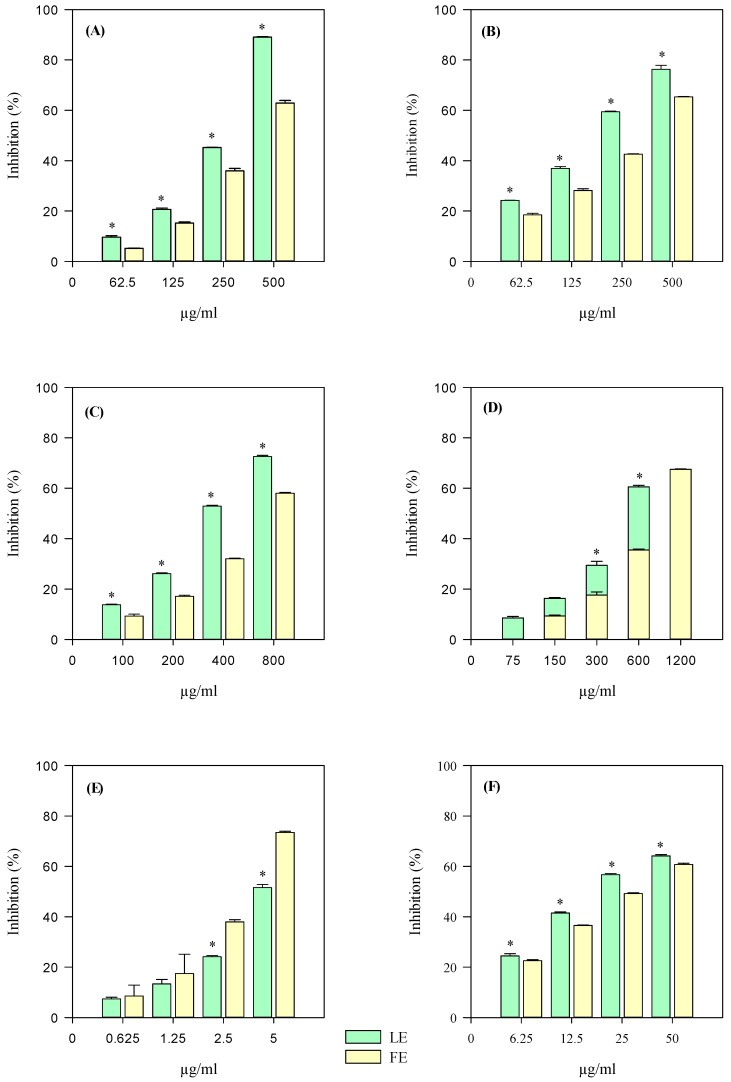
Antioxidant and free radical-scavenging concentration-dependent behaviour of *P. rubra* ‘Tonda Palermitana’ flower (yellow bars) and leaf (green bars) extracts (FE and LE, respectively) evaluated by FRAP (panel (**A**)), TEAC (panel (**B**)), DPPH (panel (**C**)), ICA (panel (**D**)), ORAC (panel (**E**)), and BCB (panel (**F**)) assays. The results, expressed as inhibition percentage (%), show the mean ± standard deviation of three independent experiments in triplicate (*n* = 3). * *p* < 0.05 vs. FE by one-way ANOVA followed by Student–Newman–Keuls Method.

**Figure 8:**
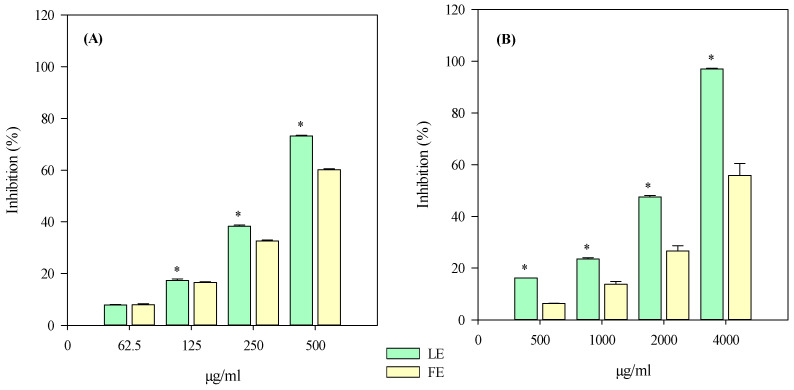
Anti-inflammatory concentration-dependent behaviour of *P. rubra* ‘Tonda Palermitana’ flower (yellow bars) and leaf (green bars) extracts (FE and LE, respectively) evaluated by PIA (panel (**A**)) and ADA (panel (**B**)) assays. The results, expressed as inhibition percentage (%), show the mean ± standard deviation of three independent experiments in triplicate (*n* = 3). * *p* < 0.05 vs. FE by one-way ANOVA followed by Student–Newman–Keuls Method.

Finally, by plotting the FE and LE concentrations with respect to the obtained inhibition percentages (yellow and green bars, respectively), interesting concentration-dependent antioxidant and anti-inflammatory behaviour was detected for both extracts, as shown in Figure 7 and Figure 8, respectively.

## 3. Discussion

This study focused on *P. rubra* ‘Tonda Palermitana’, a plant that in Italy is exclusive to Sicily, where it is renowned as an ornamental species or for its symbolic/ritual use. However, in different tropical areas of the world where the plant is spontaneous or has become naturalized, many other uses are known, e.g., medicinal, cosmetic and even as food [7,20]. In folk medicine, the leaves of *P. rubra* are used to treat inflammation [10] and various skin problems [12]. Latex is topically applied on blisters and sores [19]. In addition, flowers of *Plumeria* spp. are also used for cosmetic purposes [20].

Among the different pharmacological activities reported in the literature, extracts of *P. rubra* are noted for their antibacterial and anti-inflammatory activities [7].

Considering this, our investigation was conducted on leaf and flower extracts of plants growing in Sicily to verify their biological properties and their possible uses in medicinal and cosmetic applications. To characterize the plant material, a pharmacognostic study was carried out. Indeed, although different anatomical and phytochemical studies have been performed on many Apocynaceae species [21,22,23,24], limited data are available today on *P. rubra* leaf and flower extracts.

Our observations confirmed the presence of paracytic stomata, as already recorded in *Plumeria* species by Metcalfe and Chalk [25] and Toma et al. [26]. Solitary stomata, that have been previously observed in other genus belonging to the Apocynaceae family such as *Vinca minor* e *V. major* [27], were found.

According to Toma et al. [26], the leaves of *Plumeria* sp. are hypostomatic; on the contrary, in the present study, amphistomatic leaves similar to those found in another Apocynaceae, i.e., *Mandevilla coccinea* [21], were observed. Furthermore, in *P. rubra* ‘Tonda Palermitana’, both leaf surfaces showed striate cuticles, unlike *P. rubra* var. *alba* which lacks them [28]. Pluricellular non-glandular trichomes with an acute apex were present only in the rib’s closeness on the leaf lower surface. Instead, Araujo et al. [28], also studying the leaves of *P. rubra* var. *alba,* found trichomes near the veins, but they were unicellular and located on both epidermises. The presence of NGTs on the petal surface of *P. rubra* agrees with the data collected in other Apocynaceae by Basir et al. [29] in three *Hoya* species, and by Ramos et al. [30] in *Adenium obesum*. Trichomes covered with knobs or small protuberances along their entire length can be called ‘bosselated’ according to Payne [31]. The prominent knobs observed in bosselated trichomes of the *P. rubra* flower appeared similar to those described in the multicellular stalks of the capitate glandular trichomes on the inner surface of *Lavandula* spp. petals [32,33].

The *P. rubra* leaves showed a dorso-ventral mesophyll and a bi-collateral vascular bundle, which are common features in members of the Apocynaceae family [25]. In particular, a bi-collateral vascular bundle arranged in an open arc was present both in the leaf and petiole. In the leaf, two other smaller accessory vascular bundles were visible on both sides of the midrib. On the contrary, Carvalho et al. [22], in other Apocynaceae genera such as *Araujia* and *Morrenia*, and Toma et al. [26] in *Plumeria* sp., found them only in the petiole.

Leaves, petioles and petals were rich in branched laticifers with a polygonal or round shape in the transversal section, as already evidenced by Murugan and Inamdar [34] and Toma et al. [26] in the *Plumeria* genus. Moreover, laticifers of *P. rubra* ‘Tonda Palermitana’ were surrounded by a ring of parenchymatic cells, as described by Araujo et al. [28] in *P. rubra* var. *alba*.

Foliar and petiolar laticifers showed a distribution similar to that previously reported also by Murugan and Inamdar [31] in *P. alba*. The latex, commonly present in the Apocynaceae members, is rich in several chemical compounds [21,35], as confirmed by the histochemical tests performed, which showed a positive reaction for both alkaloids and lipophilic compounds.

These results were confirmed, in the present study, by LC-DAD-ESI-MS analysis of flower and leaf food-grade extracts. According to previous data, several compounds belonging mainly to the classes of iridoids, terpenoids, flavonoids, phenolics, alkaloids, fatty acids and coumarins were detected [7]. Although soil and climate factors, as well as harvesting and storage times can determine variability from the phytochemical point of view, *P. rubra* ‘Tonda Palermitana’ flower and leaf extracts contain several previously identified phytochemicals with well-established biological properties [7].

Among the most characteristic compounds of this species, several iridoids such as allamandin, plumieride, α-allamcidin, 13-*O*-caffeoylplumieride, 13-deoxyplumieride, plumieridin A, plumieridin B, protoplumericin A, protoplumericin B, plumeridoid A, plumieride *p*-Z-coumarate, and plumieride-*p*-E-coumarate were already identified in *P. rubra* bark, roots, flowers, stem bark and the whole plant [36,37,38,39,40,41]. Terpenoids were also very common constituents in *Plumeria* spp. and, according to our results, several triterpenoids such as rubrajaleelol, rubrajaleelic acid, rubrinol, arjunolic acid, oleanolic acid and β-amyrin acetate were already identified and isolated from different parts of the *P. rubra* plant such as the leaves, stem bark and flowers [41,42,43].

In accordance with what was observed in the present study, different flavonoids such as quercetin, quercitrin, rutin, narcissin, cyanidin-3-*O*-β-(2′-glucopyranosyl-*O*-β-galactopyranoside, cyanidin-3-*O*-β-galactopyranoside, rubranonoside, plumerubroside, kaempferol-3-rutinoside and quercetin 3-*O*-α-L-arabinopyranoside were identified and isolated from the leaves, flowers, stem bark and whole plant of *P. rubra* [41,44,45].

Among phenolic acids, p-*E*-coumaric acid, 3-*O*-caffeoylquinic acid and quinic acid, as well as the alkaloids plumericidine, plumerianine, plumerinine and grandine A, were already identified in *P. rubra* [38,43,46,47,48].

Finally, fatty acids including stearic acid and 34-hydroxy tetratriacontanyl ferulate, steroids such as stigmasterol and β-sitosterol, monoglycerides such as 2,3- dihydroxy propyl octacosanoate and the coumarin scopoletin were previously identified in different parts of *P. rubra* [42,43,49,50,51].

On the contrary, the present study highlights for the first time the presence of the following secondary metabolites in *P. rubra* leaves and flowers: hydroxycaffeic acid, sy-ringic acid-4-sulphate, myricetin 3-*O*-glucoside, glochiflavanoside B, 4-*O*-(3′-*O*-α-D-glucopyranosyl)-caffeoyl quinic acid, 3,5-Di-*O*-caffeoyl quinic acid, obtusidin, coumarobutsane, champalinin, stearyl dixylosyl methoxygallic acid, plume-noside and 13-*O*-caffeoyl-15-demethy-plumieride, many of which were already detected in *P. obtusa* [52,53].

Several studies have shown that different extracts of *Plumeria* sp. exert anti-inflammatory, antioxidant and free radical scavenging properties. These are mostly antioxidant in vitro studies carried out on leaf and flower methanolic extracts and anti-inflammatory in vivo studies conducted on murine models by administering leaf, flower and pod methanolic or ethanolic extracts [46,54,55]. In the first case, a direct comparison with the results obtained with food-grade extracts is possible, since many of the tests are the same as those used in the present study. For example, *P. rubra* methanolic leaf extract had already demonstrated interesting anti-peroxidase activity on a linoleic acid emulsion, which appears to be about one-third (IC_50_ 75 µg/mL) of that found in the present study (IC_50_ 22.05 µg/mL). According to our results, concentration-dependent behaviour was already highlighted in radical scavenging assays such as DPPH [54,55] with *P. acuminata* leaf extract. Furthermore, similar antioxidant behaviour to that observed in the present study was observed for *P. rubra* methanolic flower extract, which showed greater activity in the FRAP test, followed by DPPH and ICA [46].

The methanolic leaf extract of *P. rubra* at a dose of 500 mg/kg b.w. exhibited maximum anti-inflammatory effects, significantly reducing the formation of granuloma tissue induced by the cotton pellet method in Wistar albino rats and Swiss albino mice models [56]. Furthermore, at doses of 100 and 200 mg/kg b.w., it exhibited significant dose-dependent activity, suppressing carrageenan-induced paw edema in rats [57].

Bihani’s review [7] summarizes the traditional uses of *P. rubra*, many of which can be at least partly ascribed to the strong antioxidant and anti-inflammatory activity of its extracts, which were confirmed by the results of the present work carried out on flower and leaf hydroalcoholic extracts.

However, beyond the interesting antioxidant and anti-inflammatory activity found in in vitro and in vivo models, it is important to comment that toxicity studies have demonstrated the safety of these extracts. An acute toxicity assay carried out administering a single dose at six different concentrations (500–2000 mg/kg b.w.) of *P. rubra* methanolic leaf extract to six groups of Wistar albino rats and Swiss albino mice showed no deaths and no stereotypical symptoms over 72 h, with a calculated median lethal dose (LD_50_) greater than 2000 mg/kg b.w. [56]. A similar trend was observed also with *P. rubra* methanolic red flower extract on male Sprague Dawley rats, which showed no toxic signs, behavioural changes or mortality up to dose of 4000 mg/kg b.w. [46].

## 4. Materials and Methods

### 4.1. Plant Material

About 500 g of both leaves and flowers of *P. rubra* ‘Tonda Palermitana’ were collected from an old tree located in Mondello (PA, Italy) (38°11′53″ N, 13°19′44″ E, 27 m a.s.l., exposure South, flowerbed) during July 2022. The voucher specimen (100083) was stored in the SAF herbarium at the Department of Agricultural and Forest Science (University of Palermo). Fresh plant material was immediately sent to the University of Genova and to the University of Messina to be processed and analyzed.

### 4.2. Microscopical Analyses

The micro-morphological analysis of the leaf, petiole and flower was carried out on both fresh samples and samples fixed with the alcoholic-based fixative FineFIX (Milestone SRL, Sorisole, Bergamo, Italy) for 24 h at 4 °C. The leaf and petiole were hand-sectioned using a double-edged razor blade, while the petal surface was peeled off gently with fine tweezers. Sections and epidermal peels were then treated with Phloroglucinol-HCl (Merck, Darmstadt, Germany) to stain lignin, Sudan III (Merck, Darmstadt, Germany) or Dragendorff reagent (Merck, Darmstadt, Germany) to detect the presence of laticifers. All these samples were then mounted in water and observed by a Leica DM 2000 transmission light microscope (Leica Microsystems, Wetzlar, Germany) coupled with a ToupCam Digital Camera with a CMOS Sensor, 3.1 MP resolution (ToupTek Photonics, Hangzhou, China). In addition, some sections of leaf and petiole were cleared with an aqueous solution of chloral hydrate and mounted in a chloral hydrate–glycerol solution to prevent crystallization of the reagent during observations, according to Jackson and Snowdon [58].

Small pieces of fixed leaf, petiole and flower were also dehydrated through a graded ethanol series (70, 80, 90 and 100%) and critical point dried in CO_2_ (CPD, K850 2M Strumenti s.r.l., Rome, Italy). Two-sided adhesive carbon tapes were fixed on aluminium stubs, and then a small amount of the dried specimens was placed on this conductive substrate. The samples were finally covered with a 10 nm layer of gold particles and examined under a Scanning Electron Microscope VEGA3-Tescan-type LMU (Tescan USA Inc., Cranberry Twp, PA, USA), operating at an accelerating voltage of 20 kV.

### 4.3. Extraction Procedure

Fresh plant material was powdered by a blade mill (IKA^®^ A11, IKA^®^-Werke GmbH & Co. KG, Staufen, Germany) with liquid nitrogen to inhibit the enzymatic activity and preserve the native phytochemical profile. Food-grade flower and leaf extracts (FEs and LEs, respectively) were obtained according to Cornara et al. [59] by adding 100 mL of ethanol/water mixture (80:20, *v*/*v*) to ten grams of powdered plant material and sonicating in an ice bath for 10 min using a titanium probe set to 200 W and 30% amplitude (Vibra Cell™ Sonics Materials, Inc., Danbury, CT, USA). After this, samples were left to macerate under stirring in the dark at RT for 2 h. Supernatants were recovered by filtration on Whatman paper filter n. 1 within round bottom flasks and the extraction was repeated two more times. Collected supernatants were dried by a rotary evaporator (Büchi R-205, Cornaredo, Italy) in the dark at 37 °C and stored overnight in a vacuum glass desiccator with anhydrous sodium sulphate. After extraction yield calculation (77.59% ± 0.27 and 8.69% ± 0.43 for FE and LE, respectively), dry extracts were stored in burnished sealed vials with nitrogen headspace until analysis, when they were resuspended and properly diluted in the same hydroalcoholic mixture reported above.

### 4.4. Phytochemical Analyses

#### 4.4.1. Total Phenolic Compounds (TPCs)

Total phenolics were quantified according to Ingegneri et al. [60]. Briefly, 10 µL of FE and LE (0.625–5.0 mg/mL) were added to 90 µL of deionized water and mixed with 100 µL of Folin–Ciocalteu reagent. After 3 min, 100 µL 10% anhydrous sodium carbonate was added and samples were incubated in the dark at RT for 60 min, shaking every 10 min. Absorbance was read at 785 nm by using a Multiskan™ GO Microplate Spectrophotometer (Thermo Scientific, Waltham, MA, USA) against the hydroalcoholic mixture as a blank. Gallic acid was used as a reference compound (0.075–0.6 mg/mL; y = 0.0016x, R^2^ = 0.999), and the results were expressed as g gallic acid equivalents (GAEs)/100 g dry extract (DE).

#### 4.4.2. Total Flavonoid Compounds (TFCs)

Total flavonoids were quantified according to Lenucci et al. [61]. Briefly, 50 μL of FE and LE (2.5–20 mg/mL) was added to 450 μL of deionized water and 30 μL of 5% NaNO_2_. After 5 min, 60 μL of 10% AlCl_3_ was added and samples were incubated for 6 min at RT. Then, 200 microliters of 1 M NaOH and 210 μL of deionized water were added, and the samples were vortex mixed. The absorbance was recorded at 510 nm by a UV–Vis spectrophotometer UV-1601 (Shimadzu, Kyoto, Japan) against the hydroalcoholic mixture as a blank. Rutin was used as a reference standard (0.125–1.0 mg/mL; y = 0.0007x, R^2^ = 0.999), and the results were expressed as g of rutin equivalents (RE)/100 g DE.

#### 4.4.3. Vanillin Index

Flavan-3-ols as well as other flavonoids that have a free meta-oriented hydroxy group on the B ring were quantified by vanillin index assay [62]. FE and LE were diluted in 0.5 M H_2_SO_4_ (absorbance ranging from 0.2 to 0.4) and loaded onto a conditioned Sep-Pak C18 cartridge (Waters, Milan, Italy), which was then washed with 2.0 mL of 5.0 mM sulphuric acid. Samples were eluted with 5.0 mL of methanol and 1 mL of each eluate was added to 6.0 mL of 4% vanillin methanol solution and incubated at 20 °C for 10 min. Once the HCl (3 mL) was added, samples were incubated for 15 min at RT and the absorbance was recorded at 500 nm using the same instrument and blank reported in Section 4.4.2. Catechin was used as a reference standard (0.125–0.50 mg/mL; y = 0.0027x, R^2^ = 0.998). The results were expressed as g of catechin equivalents (CE)/100 g DE.

#### 4.4.4. Proanthocyanidins

Proanthocyanidins were quantified by hot acid hydrolysis [62] diluting FE and LE in 0.05 M H_2_SO_4_ (2 mL). The solutions were loaded onto conditioned Sep-Pak C18 cartridges (Waters, Milan, Italy). The proanthocyanidin-rich fractions obtained were eluted with methanol (3 mL) and collected in 100 mL round-bottom flasks shielded from light and containing 9.5 mL of absolute ethanol. After this, 12.5 mL of 300 mg/L FeSO_4_·7H_2_O hydrochloric acid solution was added and samples were left to reflux for 50 min. After cooling, the absorbance was recorded at 550 nm using the same instrument and blank reported in Section 4.4.2. The starting anthocyanins content of samples was estimated by recording the absorbance of samples prepared under the same conditions but cooled in ice instead of warmed. Cyanidin chloride (Cy) was used as reference standard (ε = 34,700) and results were expressed as g of Cy equivalents (CyE)/100 g DE.

#### 4.4.5. LC-DAD-ESI-MS Analysis

After a preliminary phytochemical screening, the FE and LE phytochemical profile was investigated by LC-DAD-ESI-MS analysis [62]. Separation was carried out at 25 °C using Luna Omega PS C18 column 150 mm × 2.1 mm, 5 µm (Phenomenex, Torrance, CA, USA). The following elution programme, using 0.1% formic acid (Solvent A) and acetonitrile (Solvent B) as the mobile phase, was used: 0–3 min, 0% B; 3–9 min, 3% B; 9–24 min, 12% B; 24–30 min, 20% B; 30–33 min, 20% B; 33–43 min, 30% B; 43–63 min, 50% B; 63–66 min, 50% B; 66–76 min, 60% B; 76–81 min, 60% B; 81–86 min, 0% B, equilibrated for 4 min. Five microliters of FE and LE were injected, recording the UV–Vis spectra from 190 to 600 nm. Acquisition was carried out at different wavelengths (260, 280, 292, 330, 370 and 520 nm) to identify all polyphenols classes. An ion trap (model 6320, Agilent Technologies, Santa Clara, CA, USA) coupled with an electrospray ionization source (ESI) operating both in negative and positive ionization mode was used by setting the parameters as follows: 3.5 kV capillary voltage, 40 psi nebuliser (N_2_) pressure, 350 °C drying gas temperature, 9 L/min drying gas flow and 40 V skimmer voltage. Acquisition was carried out in full-scan mode (90–2000 *m*/*z*). Data were acquired by Agilent ChemStation software version B.01.03 and Agilent trap control software version 6.2. Identification was carried out by comparing the retention times, UV–Vis and MS spectra of each analyte with those of commercially available standards (see Table 2), literature data and UV–Vis and open-source mass spectra databases.

### 4.5. In Vitro Antioxidant and Anti-Inflammatory Assays

The antioxidant and anti-inflammatory activity of FE and LE were evaluated by several in vitro spectrophotometric and spectrofluorimetric assays based on different mechanisms and reaction environments. The results were expressed as inhibition (%) of the oxidative/inflammatory activity by calculating the half-inhibitory concentration (IC_50_) and the respective confident limits (C.L.s) at 95% by Litchfield and Wilcoxon’s test (PHARM/PCS 4, MCS Consulting, Wynnewood, PA, USA). The following reported concentration ranges refer to final concentrations in the reaction mixture.

#### 4.5.1. DPPH

The reaction mixture, consisting of FE or LE (62.50–500 µg/mL) and 2.50 mg/mL fresh DPPH methanol solution (1:40, *v*/*v*), was mixed and incubated in the dark at RT for 20 min [60]. The absorbance was recorded at 517 nm using the same instrument and blank reported in Section 4.4.1. Trolox was used as a reference standard (2.5–20.0 μg/mL; y = −0.0008x + 0.7428, R^2^ = 0.999).

#### 4.5.2. TEAC

The blue-green cationic radical solution, obtained by incubating at RT for 12 h, the 1.7 mM diammonium salt of 2,20-azino-bis (3-ethylbenzothiazolin-6-sulphonic acid (ABTS) with 4.3 mM K_2_S_2_O_8_, was diluted with deionized water to obtain an absorbance of 0.7 ± 0.02 at 734 nm and used within 4 h. Then, 10 microliters of FE and LE (31.25–250.0 µg/mL) were added to the radical solution (200 μL) and incubated at RT for 6 min [60]. The absorbance decrease was recorded at 734 nm using the same instrument and blank reported in Section 4.4.1. Trolox was used as a reference standard (1.25–10.0 µg/mL; y = −0.0031x + 0.693, R^2^ = 0.999).

#### 4.5.3. FRAP

FE and LE (62.5–500.0 µg/mL) were added to fresh pre-warmed (37 °C) working reagent (1:20, *v*/*v*), consisting of 300 mM buffer acetate (pH 3.6), 10 mM 2,4,6-Tris(2-pyridyl)-s-triazine (TPTZ) dissolved in 40 mM HCl and 20 mM iron(III) chloride, and incubated for 4 min at RT in the dark [60]. The absorbance was recorded at 593 nm using the same instrument and blank reported in Section 4.4.1. Trolox was used as a reference standard (1.25–10.0 μg/mL; y = 0.053x, R^2^ = 0.999).

#### 4.5.4. ORAC

FE and LE (1.25–10.0 µg/mL and 0.625–5.0 µg/mL, respectively) were added to fresh 117 nM fluorescein solution and incubated for 15 min at 37 °C. After this, 40 mM 2,2′-Azobis(2-methylpropionamidine) dihydrochloride (AAPH) solution was added, achieving the following reagents ratio (1:6:3 *v*/*v*/*v*, respectively) [60]. The fluorescein decay was recorded every 30 s for 90 min (λ_ex_ 485; λ_em_ 520) by a microplate reader (FLUOstar Omega, BMG LABTECH, Ortenberg, Germany) using the same blank reported in Section 4.4.1. Trolox was used as a reference standard (0.25–2.0 μg/mL; y = 418.61x, R^2^ = 0.999).

#### 4.5.5. BCB

The BCB assay was carried out according to Cornara et al. [59] with some modifications. Briefly, 80 µL of FE and LE (6.25–50.0 µg/mL) were added to β-carotene emulsion consisting of 250 µL β-carotene chloroform solution (1 mg/mL), 1.6 µL linoleic acid, 16 µL Tween-40 and 2 mL of air-insufflated deionized water. A β-carotene free emulsion was used as a negative control, whereas a β-carotene emulsion with hydroalcoholic mixture was used as a blank. Samples were incubated for 120 min at 50 °C in a shaking water bath, monitoring the absorbance decay every 20 min at 470 nm using the same instrument reported in Section 4.4.1. Butylhydroxytoluene (BHT) was used as reference standard (0.06–0.5 µg/mL; y = 0.8376x, R^2^ = 0.999).

#### 4.5.6. ICA

The iron-chelating activity was evaluated according to Cornara et al. [59] with some modifications. Briefly, 25 µL of 2.0 mM iron (II) chloride tetrahydrate was added to 50 µL of FE and LE (0.150–1.2 mg/mL and 0.075–0.6 mg/mL, respectively) and incubated at RT for 5 min. Then, 50 µL of 5 mM ferrozine was added and the reaction mixture was diluted to 1.5 mL with deionized water, vortex mixed, and incubated for 10 min at RT. The absorbance was read at 562 nm using the same instrument and blank reported in Section 4.4.2. EDTA was used as a reference standard (1.5–12.0 µg/mL; y = −0.0009x + 0.4161, R^2^ = 0.998).

#### 4.5.7. ADA

FE and LE (0.500–4.0 mg/mL) were added to 0.4% fatty-acid-free BSA solution and PBS pH 5.3 (4:5:1 *v*/*v*/*v*, respectively) [63]. Once the starting absorbance was recorded at 595 nm, samples were incubated for 30 min at 70 °C in a shaking water bath, recording the final absorbance at the same wavelength using the same instrument and blank reported in Section 4.4.1. Diclofenac sodium was used as a reference standard (3.0–24.0 µg/mL; y = −0.012x + 1.049, R^2^ = 0.999).

#### 4.5.8. PIA

In this step, 20 microliters of FE and LE (62.50–500.0 µg/mL) were added to 12 µL of trypsin (10 µg/mL), 188 µL of Tris-HCl buffer pH 7.5 (25 mM) and 400 µL of casein (0.8%) and incubated for 20 min at 37 °C in a shaking water bath [63]. Perchloric acid (400 µL) was added to stop the reaction. After centrifugation (3500× *g* for 10 min), the absorbance of the supernatants was recorded at 280 nm using the same instrument and blank reported in Section 4.4.2. Diclofenac sodium was used as a reference standard (2.0–16.0 µg/mL; y = −1.2843x, R^2^ = 0.998).

### 4.6. Statistical Analysis

Data were expressed as IC_50_ with respective 95% C.L. (see Section 4.5) or as mean ± S.D. of three independent experiments in triplicate (*n* = 3). The statistical significance was evaluated using one-way analysis of variance (ANOVA) followed by the Student–Newman–Keuls method. Values of *p* < 0.05 were considered statistically significant. Data analysis was performed with SigmaPlot 12.0 software (Systat Software Inc., San Jose, CA, USA). Chemometric analysis, such as dendrogram and hierarchical clustering analysis (HCA), was carried out by JMP7 (SAS Institute Inc., Cary, NC, USA). To compare the differences between FE and LE, the Euclidean distance was taken, and a hierarchical clustering analysis was carried out using Ward’s variance-minimizing method.

## 5. Conclusions

This is the first study which investigated the old Sicilian cultivar *P. rubra* ‘Tonda Pa-lermitana’. The multidisciplinary approach used allowed us to conduct a complete pharmacognostic evaluation of this plant species, from micro-morphological to phytochemical and finally biological characterization. Some peculiar micro-morphological features of this ancient cultivar that have not been found before in other species belonging to the *Plumeria* genus were documented, as well the presence of some phytochemicals that have been identified for the first time in this plant species. Finally, the preparation of flower and leaf food-grade extracts has allowed us to evaluate the antioxidant and anti-inflammatory activities. This proves, for the first time, that the most interesting part of the plant from the biological point of view is the leaves. The overall data collected allow us to speculate that, beyond the scent of its splendid flowers, whose essential oil is widely used today in the cosmetic and pharmaceutical industries, *P. rubra* ‘Tonda Palermitana’ flower and leaf food-grade extracts represent very interesting plant complexes that could certainly find industrial applications. The anti-inflammatory properties and especially the high antioxidant power found for this species are promising for the treatment of free radical damage, which has a central role in the onset and development of ageing and related diseases. Therefore, considering the great interest of the industry in using extracts rich in natural antioxidants, our data allow us to assume future applications of *P. rubra* hydroalcoholic extracts for the formulation of nutraceutical, cosmetic or dermatological products with anti-ageing effects.

## Figures and Tables

**Table 1:** Phytochemical screening of *P. rubra* ‘Tonda Palermitana’ flower and leaf extracts (FE and LE, respectively). Results are the mean ± standard deviation (S.D.) of three independent experiments in triplicate (*n* = 3).

Phytochemical Assay	FE	LE
Total phenols (g GAE ^a^/100 g DE ^b^)	2.234 ± 0.066	2.696 ± 0.125 *
Flavonoids (g RE ^c^/100 g DE)	1.462 ± 0.054	1.879 ± 0.082 *
Vanillin index (g CE ^d^/100 g DE)	0.228 ± 0.012	0.317 ± 0.021 *
Proanthocyanidins (g CyE ^e^/100 g DE)	0.002 ± 0.000	0.002 ± 0.000
Polimerization index ^f^	114.00	158.50

^a^ GAE, gallic acid equivalents; ^b^ DE, dry extract; ^c^ RE, rutin equivalents; ^d^ CE, Catechin equivalents; ^e^ CyE, Cyanidin equivalents; ^f^ Polymerization index = vanillin index/proanthocyanidins. * *p* < 0.05 vs. FE.

**Table 2:** Phytochemical profile of *P. rubra* ‘Tonda Palermitana’ flower and leaf extracts (FE and LE, respectively) tentatively identified by LC-DAD-ESI-MS using both the positive and negative ionization modes.

Compound Name	RT ^c^ (min)	Molecular Formula	Molecular Weight	[M − H]^−^ (*m*/*z*)	[M + H]^+^ (*m*/*z*)	FE ^d^	LE ^e^
Hydroxycaffeic acid	9.8	C_9_H_8_O_5_	196	195		−	+
Syringic acid-4-sulphate	13.3	C_9_H_10_O_8_S	278	277		−	+
Myricetin 3-*O*-glucoside ^a^	14.6	C_21_H_20_O_13_	480	479		+	+
Plumerubroside	15.5	C_24_H_30_O_12_	511		512	+	+
Quinic acid ^a^	16.2	C_7_H_12_O_6_	192	191		+	−
Glochiflavanoside B	17.5	C_24_H_30_O_12_	510	509	511	−	+
4-Coumaric acid ^a^	19.1	C_9_H_8_O_3_	164	163		−	+
α-allamcidin	19.3	C_15_H_18_O_7_	310	309		+	−
Kaempferol-3-rutinoside ^a^	20.6	C_27_H_30_O_15_	595	594	569	+	−
13-deoxylplumieride	20.8	C_21_H_26_O_11_	454	453		−	+
Quercetin-3-rutinoside (Rutin) ^a^	21.1	C_27_H_30_O_16_	610	609		+	−
Quercetin 3-*O*-α-L-arabinopyranoside	21.5	C_20_H_18_O_11_	434	433		−	+
4-*O*-(3′-*O*-α-D-glucopyranosyl)-Caffeoyl quinic acid	22.8	C_22_H_28_O_14_	516	515		+	+
3,5-Di-O-caffeoyl quinic acid ^a^	24.5	C_25_H_24_O_12_	516	515		−	+
Quercetin-3-*O*-pentosylpentoside	25.0	C_25_H_26_O_15_	566		567	+	−
Obtusidin	26.0	C_15_H_16_O_5_	276	275	277	−	+
3-*O*-caffeoylquinic acid (Chlorogenic acid) ^a^	26.3	C_16_H_18_O_9_	354	353	355	+	−
Coumarobtusane	26.6	C_39_H_58_O_5_	607	606	608	−	+
Quercetin 3-rhamnoside (Quercitrin) ^a^	27.6	C_21_H_20_O_11_	448	447		+	−
Cyanidin-3-*O*-(2″-*O*-β-glucopyranosyl-β-glucopyranoside)	29.8	C_27_H_31_O_16_	611	610	612	+	−
15-demethy-plumieride *p*-E-coumarate	31.0	C_29_H_29_O_14_	602	601		−	+
Grandine A	31.2	C_17_H_13_NO_4_	318	317		+	−
13-*O*-caffeoylplumieride	32.9	C_30_H_32_O_15_	632		633	+	−
α-Protoplumericin B	33.7	C_35_H_39_O_19_	764	763	765	+	−
*p*-methoxy-*E*-cinnamoyloxy plumieride (Champalinin)	34.1	C_31_H_34_O_14_	648	647	649	−	+
Plumieride ^b^	35.0	C_21_H_26_O_12_	470		471	+	−
Scopoletin ^a^	36.0	C_10_H_8_O_4_	524	523	525	+	−
Rubranonoside	36.6	C_27_H_31_O_14_	580	579		−	+
Isorhamnetin-3-*O*-rutinoside (Narcissin) ^a^	37.3	C_28_H_32_O_16_	625	624	626	+	+
Protoplumericine A ^b^	39.0	C_36_H_42_O_19_	778	777		+	−
Plumeridoid A	39.5	C_14_H_16_O_7_	296	295		+	−
Plumericidine/Plumieranine	40.2	C_13_H_13_NO_3_	231	230	232	+	+
Stearyl dixylosyl methoxygallic acid	41.4	C_36_H_58_O_15_	730	729	731	+	+
Plumieride *p*-Z-coumarate	43.1	C_30_H_32_O_14_	617	616		+	+
Rubrinol	43.7	C_30_H_50_O_2_	443		444	+	+
Plumieride-*p*-E-coumarate	44.3	C_30_H_32_O_14_	617	616	618	+	+
Plumieridin A	45.8	C_13_H_16_O_5_	252		253	+	+
Allamandin	46.1	C_15_H16O_7_	308	307		+	−
Plumieridin B	47.3	C_13_H_16_O_5_	252		253	+	+
Rubrajaleelic acid	47.9	C_29_H_46_O_4_	458	457		−	+
Oleanolic acid ^a^	48.4	C_30_H_48_O_3_	457	456		−	+
β-Sitosterol 3-*O*-glucoside	49.7	C_35_H_60_O_6_	577		578	+	+
2,3- dihydroxy propyl octacosanoate	50.9	C_31_H_62_O_4_	499	498	500	+	−
β-amyrin acetate ^b^	51.4	C_32_H_52_O_2_	469	468		−	+
Methyl stearate ^b^	61.7	C_19_H_38_O_2_	299		300	+	+
Rubrajaleelol	52.2	C_29_H_48_O_3_	444		445	+	−
Stearic acid ^b^	53.3	C_18_H_36_O_2_	284		285	+	−
Stigmasterol ^b^	55.1	C_29_H_48_O	413		414	−	+
Plumerinine	56.3	C_14_H_27_NO	225		226	+	+
Plumenoside	58.6	C_20_H_24_O_11_	440		441	−	+
Arjunolic acid ^b^	64.2	C_30_H_48_O_5_	489		490	+	+
Quercetin ^a^	74.8	C_15_H_10_O_7_	302		303	−	+
13-*O*-caffeoyl-15-demethy-plumieride	78.6	C_35_H_39_O_20_	780		781	−	+
34-hydroxy tetratriacontanyl ferulate	79.1	C_44_H_78_O_5_	687		688	+	−

^a,b^ Check with commercially available HPLC-grade reference standards (purity ≥ 98%) purchased from Extrasynthase (Genay, France) and Merck KGaA (Darmstadt, Germany), respectively; ^c^ RT, retention time; ^d^ FE, *P. rubra* flower dry extract; ^e^ LE, *P. rubra* leaf dry extract; −, not found; +, found.

**Table 3:** Antioxidant and anti-inflammatory activity of *P. rubra* ‘Tonda Palermitana’ flower and leaf extracts (FE and LE, respectively) in comparison with the reference standards. The results, which represent the mean of three independent experiments in triplicate (*n* = 3), are expressed as the concentration inhibiting 50% of the oxidant/inflammatory activity (IC_50_) with 95% confidence limits (between brackets).

Test	FE	LE	RS ^a^
	µg/mL		
DPPH	689.92 (568.48–785.08)	378.26 (305.55–505.27) *	8.96 (4.80–16.72) ***
TEAC	293.54 (225.27–328.12)	210.53 (155.88–248.82)	4.10 (2.19–7.67) ***
FRAP	397.56 (295.44–492.65)	280.80 (185.43–297.64)	3.67 (1.53–8.82) ***
ORAC	6.77 (4.65–8.38)	4.86 (2.37–6.89)	0.75 (0.25–0.98) ***
BCB	25.42 (12.12–37.88)	22.05 (11.56–35.01)	0.42 (0.23–0.62) ***
ICA	889.23 (822.52–965.08)	495.81 (418.42–595.18) *	5.54 (2.30–7.38) ***
ADA	3579.78 (3214.55–3876.23)	2104.02 (1802.27–2576.08) *	39.24 (27.56–55.29) ***
PIA	382.68 (349.65–401.34)	326.23 (287.63–365.18)	35.35 (21.95–56.93) ***

^a^ RS, Reference standard: trolox for FRAP, DPPH, TEAC, and ORAC assay; BHT for β-carotene bleaching assay; diclofenac sodium for anti-inflammatory assays (ADA and PIA); * *p* < 0.05 vs. FE; *** *p* < 0.001 vs. FE and LE.

## Data Availability

The original contributions presented in the study are included in the article; further inquiries can be directed to the corresponding authors.
